# Differential Migration of Mesenchymal Stem Cells to Ischemic Regions after Middle Cerebral Artery Occlusion in Rats

**DOI:** 10.1371/journal.pone.0134920

**Published:** 2015-08-04

**Authors:** Soo Hyun Lee, Kyung Sil Jin, Oh Young Bang, Byoung Joon Kim, Soo Jin Park, Na Hee Lee, Keon Hee Yoo, Hong Hoe Koo, Ki Woong Sung

**Affiliations:** 1 Samsung Genome Institute, Samsung Medical Center, Sungkyunkwan University School of Medicine, Seoul, Republic of Korea; 2 Department of Pediatrics, Samsung Medical Center, Sungkyunkwan University School of Medicine, Seoul, Republic of Korea; 3 Department of Neurology, Samsung Medical Center, Sungkyunkwan University School of Medicine, Seoul, Republic of Korea; Hungarian Academy of Sciences, HUNGARY

## Abstract

To evaluate the optimal timing of mesenchymal stem cell (MSC) transplantation following stroke, rats were transplanted with MSCs at 1 (D1), 4 (D4), and 7 days (D7) after middle cerebral artery occlusion (MCAo). Rats in the D1 group showed a better functional recovery than those in the D4 or D7 groups after MCAo. MSCs preferentially migrated to the cortex in the D1 group, while the MSCs in the D4 or D7 groups preferentially migrated to the striatum. Interestingly, the level of monocyte chemotactic protein-1 (MCP-1) in the cortex was highest at 1 day after MCAo, while the level of stromal cell-derived factor-1 (SDF-1) in the striatum was lowest at 1 day after MCAo and then increased over time. The pattern of MCP-1 and SDF-1 level changes according to the time after MCAo was consistent with *in vivo* and *in vitro* migration patterns of MSCs. The results suggest that an earlier MSC transplantation is associated with a better functional recovery after stroke, which could be explained by the preferential migration of MSCs to the cortex in the early transplantation group. The time-dependent differential expression of MCP-1 and SDF-1 between ischemic regions seemed to mediate the differential migration of MSCs. Highest level of MCP-1 at one day of stroke may induce preferential migration of MSCs to the cortex, then better functional improvement.

## Introduction

Mesenchymal stem cells (MSCs) have been viewed as potential candidates for cell therapy in cases of ischemic stroke since it was known that MSCs derived from bone marrow (BM) can differentiate into neuronal and glial lineage cells [1]. Researches has indicated that transplanting MSCs after cerebral ischemia induction can reduce infarct size and improve functional outcomes in animal cerebral ischemia models [2–4]. The proposed mechanisms for these beneficial effects of MSCs include neuroprotection, angiogenesis, and stimulation of neurogenesis and synaptogenesis [5–8].

Investigators have explored the effect of MSC transplantation on functional recovery in animal models at various time points after stroke [9–11]. In the first clinical study by Bang et al., patients were transplanted with autologous BM-derived MSCs at 4–5 and 7–9 weeks after the onset of stroke [12]. Patients who were given MSC transplants showed a greater functional recovery than the control group, and there were no deaths, stroke recurrences, or serious adverse effects in the treatment group. In this trial, BM-derived plastic-adherent cells had to be expanded in culture for long time to get the target cell number (1 × 10^8^ cells/patient). The time interval between the onset of stroke and MSC transplantation may be important in terms of the efficacy of MSC transplantation for stroke. However, there has been no consensus for the optimal time of MSC transplantation following a stroke. For this reason, this study evaluated whether there were a significant time-dependent differences in the efficacy of MSC transplantation after an ischemic stroke.

## Materials and Methods

### MSC Preparation

MSCs were obtained from bone marrow of adult male Sprague-Dawley (SD) rats (n = 20). Mononuclear cells were isolated from bone marrow aspirates using Ficoll-Hypaque density gradient centrifugation (Histopaque-1077; Sigma–Aldrich, St. Louis, MO). Cells were seeded at a density of 3 × 10^5^ cells/cm^2^ in Dulbecco’s Modified Eagle Medium (DMEM; No. 11885, GIBCO Invitrogen, Carlsbad, CA) supplemented with 10% fetal bovine serum (FBS; No. 26140, GIBCO Invitrogen, Carlsbad, CA), 1% penicillin, and 1% streptomycin. The cells were incubated in a humidified atmosphere at 37°C with 5% CO_2_ and the medium was changed every 3 to 4 days until the adherent fibroblast-like cells reached confluence. Plastic-adherent MSCs were collected and resuspended in fresh culture medium and transferred to new flasks and then sub-cultured. These cells were uniformly positive for CD29, CD44, CD73, CD90, CD105 and CD166. In contrast, they were negative for the hematopoietic lineage markers CD34, CD45, CD14 and HLA–DR as confirmed by flow cytometric analysis of expressed surface antigens. These MSCs differentiate into cells for osteogenic, chondrogenic and adipogenic lineages. The MSCs used in the present study were harvested at passage 5.

### Animal Model Surgical Preparation

All animal procedures were approved by the Institutional Animal Care and Use Committee (IACUC) of Samsung Biomedical Research Institute (SBRI) (permit number: C-B1-113-2) and performed in accordance with the Institute of Laboratory Animal Resources (ILAR) guide. All animals were maintained in a facility accredited by the Association for Assessment and Accreditation of Laboratory Animal Care International (AAALAC #001003). SD rats (8 weeks old, 270–300 g) were initially anesthetized with 3.5% isoflurane and maintained with 1.0–2.0% isoflurane in 2:1 N_2_O:O_2_ using a face mask. Transient middle cerebral artery occlusion (MCAo) was induced using an intraluminal vascular occlusion method [13]. To detect the occlusion or reperfusion and the unintentional generation of subarachnoid hemorrhage during thread occlusion of the middle cerebral artery, a single laser Doppler flowmetry (LDF, Probe 418, Perimed, Stockholm, Sweden) probe was positioned over the ipsilateral cortex. One and a half hours after MCAo, the animals were reanesthetized with isoflurane and reperfusion was performed by withdrawing the suture. Triphenyltetrazolium chloride (TTC) dye test was performed to confirm the presence of infarction in randomly selected rats. Rats with 9–12 points of the modified Neurological Severity Score (mNSS) on both day 0 and day 1 of stroke were used in this study. A sham-operated control group was treated in an identical fashion with the omission of vascular occlusion. The mortality rate of rats was 30% during the operation.

### MSC Transplantation

**Fig 1:**
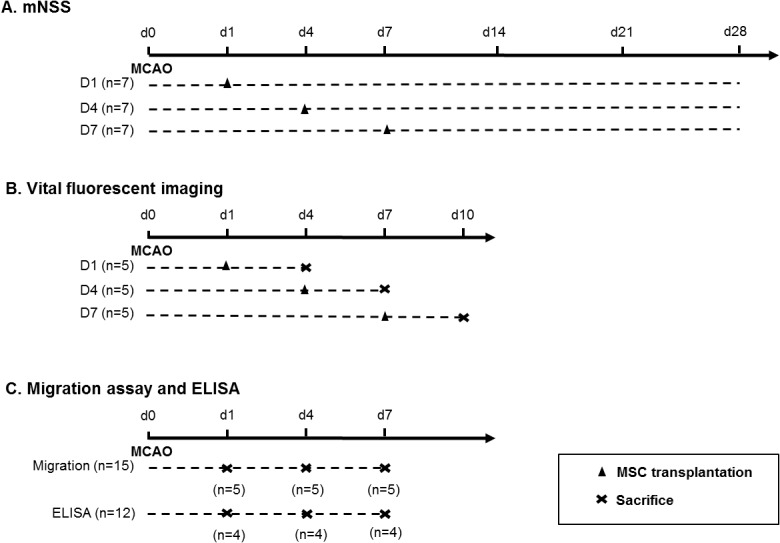
Study design. MSCs were transplanted to the experimental groups at 1, 4, and 7 days after MCAo. Each of these three groups was referred to as the D1, D4, and D7 groups. mNSS indicates modified neurological severity score; MCAo, middle cerebral artery occlusion.

Approximately 3 × 10^6^ MSCs in 600 μL total fluid volume were given via intravenous injection using multi-syringe infusion/withdrawal pump (KDS 230, Seoul, Korea, 200 μL/min) to the experimental groups at 1, 4, and 7 days after MCAo. Each of these three groups were referred to as the D1, D4, and D7 groups (n = 7 per group). One mL PBS was given as an injection to the PBS control group at 1 day after MCAo. The mortality rate of rats was 30% during the transplantation. The study design is shown in Fig 1.

### Neurological Function Tests

A battery of functional tests was performed prior to MCAo and at 1, 4, 7, 14, 21, and 28 days after the MCAo procedure. The mNSS was used to assess the procedures, and neurological function was graded on a scale of 0 to 14 (normal score 0, maximal deficit score 14). Originally, NSS is determined as a composite of motor, sensory, reflexes, and balance tests [14]; however, this study did not score the NSS reflex responses to exclude ambiguous determination. Rats with 9–12 mNSS points on both day 0 and day 1 of stroke were used in this study.

### Vital Fluorescent Imaging

Chloromethylbenzamido-1,1'-dioctadecyl-3,3,3',3'-tetramethylindocarbocyanine perchlorate (CM-DiI; No. C-7000, CellTracker, Molecular Probes, Eugene, OR) was used according to the manufacturer’s instructions. MSCs were stained for 5 minutes at 37°C in CM-DiI working solution and then for an additional 15 minutes at 4°C. As a control, unstained MSCs went through the same process without the use of the CM-DiI working solution. Both stained and unstained MSCs were then resuspended in 600 μL PBS and 3 × 10^6^ MSCs were intravenously transplanted at 1, 4, and 7 days after MCAo (n = 5 for each group). Unstained MSCs were also transplanted at 1 day after MCAo (n = 2). Rats were sacrificed 3 days after MSC transplantation and CM-DiI fluorescent signals were analyzed both on the whole brain surface and with 2 mm thickness slices using a Xenogen IVIS system (Xenogen Corporation, Alameda, CA). Rats were transcardially perfused with PBS followed by 4% paraformaldehyde. After decapitation, the brains were removed and kept in 4% paraformaldehyde solution overnight at 4°C, and then placed in 30% sucrose solution. The brains were cut into 20-micron cryostat sections. The tissues were mounted in Vectashield mounting media with 4',6-diamidino-2-phenylindole (DAPI) (Vector Laboratories, Burlingame, CA) and viewed using the Carl Zeiss LSM 700 confocal microscopy system (Jena, Germany), operated with appropriate wavelengths to detect DAPI staining of nuclei and CM-DiI-labeled MSCs. Confocal images were analyzed using LSM 700 Zen software.

### In Vitro Migration Assay

Rats were sacrificed at 1, 4, and 7 days after MCAo (n = 5 at each time point) and the ischemic cortical and striatal regions were immediately extracted. The brain segments were homogenized by adding DMEM (150 mg tissue/mL DMEM) and were incubated on ice for 10 minutes. The homogenate was centrifuged at 10,000 *g* for 20 minutes at 4°C and the supernatant was stored at −70°C until the beginning of the experiment. Brain extract was diluted to 20% in serum-free DMEM immediately prior to use.

In vitro migration was evaluated using a 24-well plate and a transwell system (8 μm pore, Corning Life Sciences) [15]. For migration assays, the lower side of the transwell filter was coated with 50 μg/mL fibronectin (Sigma) for 1 hour at 37°C. After a 6-hour starvation in serum-free DMEM, 5 × 10^4^ MSCs resuspended in 100 μL serum-free DMEM were placed in the upper chamber and 600 μL of serum-free DMEM with or without 20% brain extract were placed in the lower chamber. After a 4-hour incubation period, the transwell filter was removed and washed with PBS. After cleaning the upper face of the transwell filter with a cotton swab, MSCs in the lower face of filter were stained with hematoxylin (MHS16; Sigma, St. Louis, MO) and eosin (HST110216; Sigma, St. Louis, MO) (H&E). The number of stained MSCs was counted manually in 5 fields under a light microscope with 100-fold magnification.

### ELISA

Quantitative immunoassays using ELISA (R&D Systems Inc., Minneapolis, MN) were performed to analyze the expression level of monocyte chemotactic protein-1 (MCP-1) and stromal cell-derived factor-1 (SDF-1) in brain extracts obtained from ischemic cortical and striatal regions at 1, 4, and 7 days after MCAo (n = 4 for each group). The brain extracts from sham-operated rats were used as a control. The samples were prepared according to the manufacturer’s instructions, and then the optical density (OD) was read on a spectrophotometer and the experimental data that were obtained were compared with a standard MCP-1 and SDF-1 curve using a SoftMax pro 4.8 program (Molecular Devices, Sunnyvale, CA).

### Statistical Analysis

All data from functional tests, imaging, migration assays, and ELISA were expressed as mean ± standard deviation (SD). Data were analyzed by repeated-measures analysis of variance and unpaired Student’s t-test in SPSS version 16, if they have normal distribution (Kolmogorov-Smirov test, *P* > 0.05). Two-tailed probability values of < 0.05 were considered statistically significant.

## Results

### Neurological Function Test

**Fig 2:**
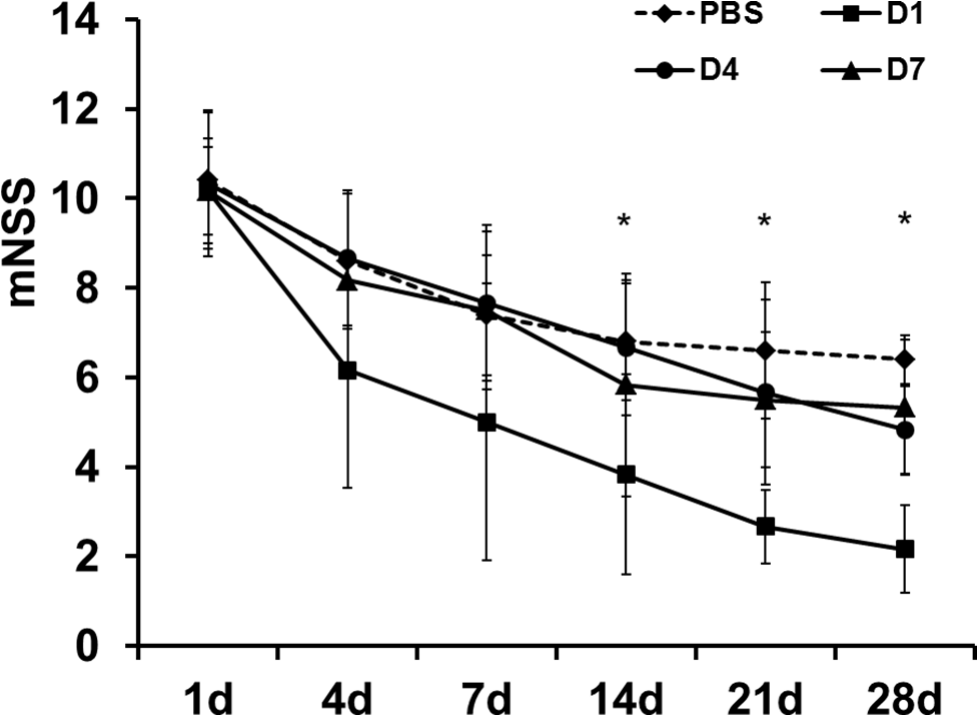
Modified Neurological Severity Score (mNSS) results according to the time after MCAo. The mNSS was lower in the D1 group compared to the control (PBS group), D4, or D7 groups at each time point 14 days after MCAo (*P < 0.05) (n = 7 per group). MCAo indicates middle cerebral artery occlusion.

No difference was observed in the mNSS evaluation between groups at day 0 and day 1 of MCAo. Prominent functional improvement was observed in the D1 group compared to the control (PBS group), D4, or D7 groups at each time point 14 days after MCAo (Fig 2). The D4 and D7 groups also showed a functional improvement trend compared to the control group; however, it was not statistically significant (n = 7 per group).

### MSC Distribution According to Time of Transplantation

**Fig 3:**
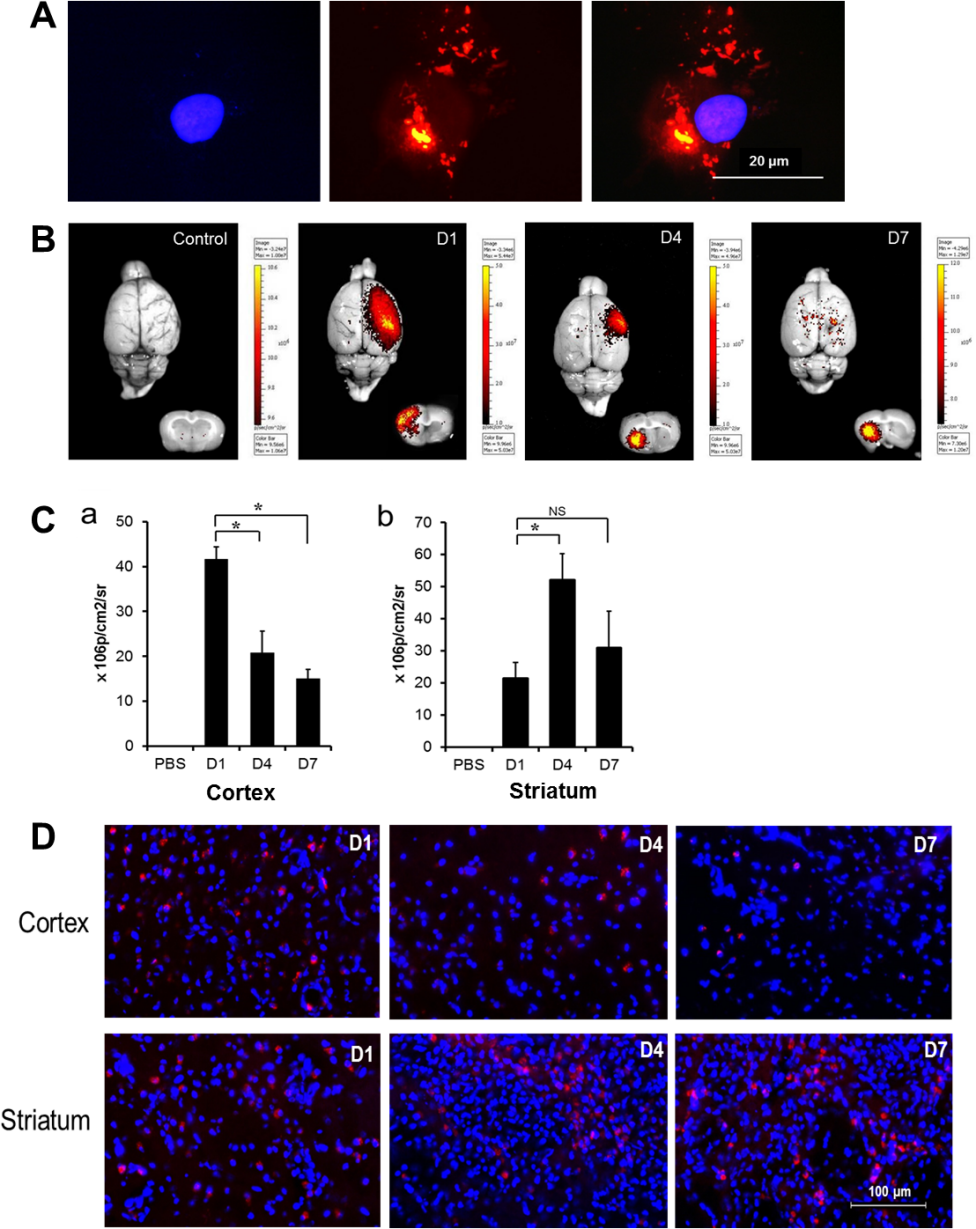
Fluorescent imaging. (A) Vital fluorescent dye staining with CM-Dil did not affect the viability and morphology of the MSCs. (B) Control group transplanted with unstained MSCs showed no fluorescent signals both on the whole brain surface and in slices. Fluorescent signals on the surface of the MCA territory or cortical region of slices were stronger in the D1 group than in the D4 or D7 groups. However, fluorescent signals on the striatal region of slices were weaker in the D1 group than in the D4 or D7 groups. (C) The quantification of MSCs by photon emission (photons/s/cm^2^/sr) representing fluorescence intensity also showed similar results (*P < 0.05) (n = 5 for each group). (D) The MSCs stained with CM-DiI were dominantly detected at the cortex in the D1 group and at striatum in the D4 and D7 groups. CM-DiI indicates chloromethylbenzamido-1,1'-dioctadecyl-3,3,3',3'-tetramethylindocarbocyanine perchlorate; MSCs, mesenchymal stem cells; MCA, middle cerebral artery.

Vital fluorescent dye staining with CM-Dil did not affect the viability and morphology of the MSCs (Fig 3A). The vital fluorescent imaging study indicated that the fluorescent signals on the cortex were stronger in the D1 group than in the D4 or D7 groups. However, fluorescent signals on the striatum were weaker in the D1 group than in the D4 or D7 groups (Fig 3B). The quantification of MSCs by photon emission (photons/s/cm^2^/sr) also showed that the fluorescent intensity in the cortex was highest in the D1 group. In comparison, in the striatum, the intensity was highest in the D4 group (Fig 3C). Similarly, the microscopic examination of MSCs stained with CM-DiI indicated that the MSCs were predominantly observed at the cortex in the D1 group, but were predominantly observed at the striatum in the D4 and D7 groups (Fig 3D). CM-DiI labelled MSCs maintained their regular cellular morphology (Fig 3D).

### In Vitro Migration Assay

**Fig 4:**
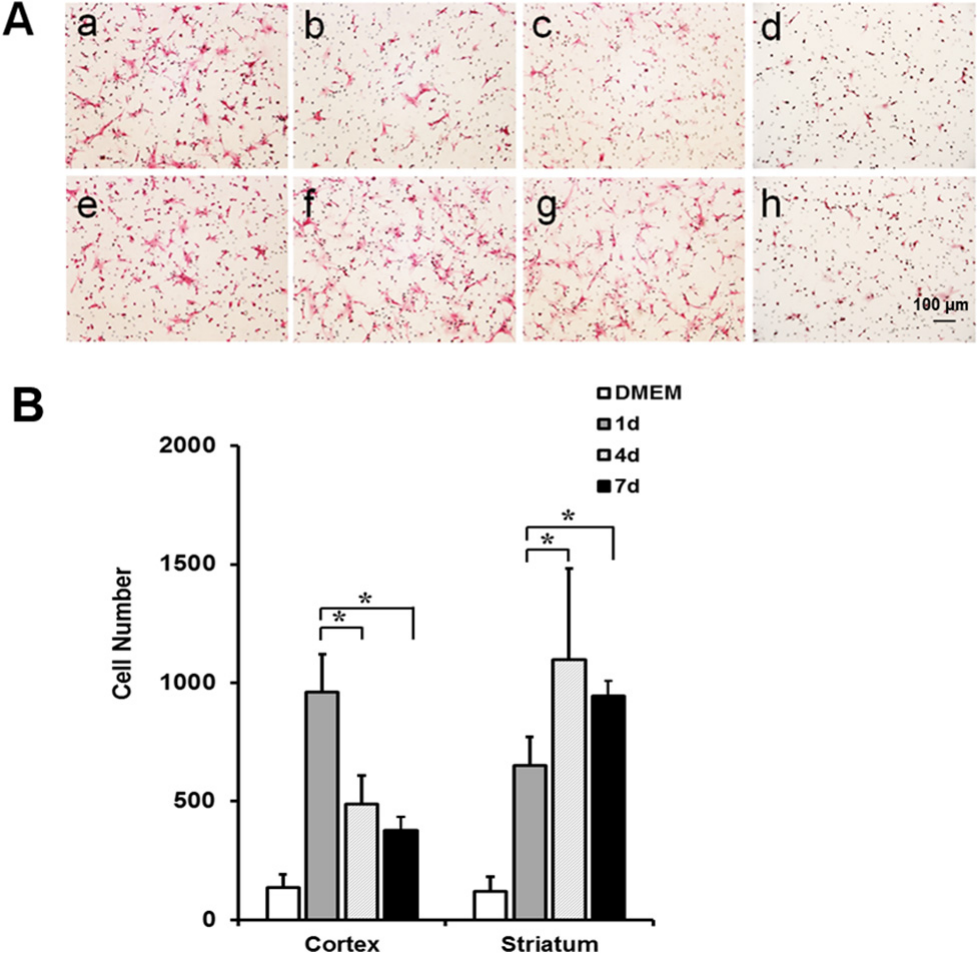
*In vitro* migration assay using a transwell system with brain extracts obtained from cortex and striatum at 1, 4, and 7 days after MCAo. (A and B) Transwell migration of MSCs with brain extracts obtained from ischemic region (a-c and e-f) was more prominent than without brain extract (d and h). Transwell migration of MSCs was more prominent with brain extract obtained from cortex at 1 day after MCAo (a) than with those obtained at 4 (b) or 7 (c) days after MCAo. However, transwell migration with brain extract obtained from striatum at 1 day after MCAo (e) was lower than with those obtained at 4 (f) or 7 (g) days after MCAo (*P < 0.05) (n = 5 for each time point). MSCs indicates mesenchymal stem cells; MCAo, middle cerebral artery occlusion.

We hypothesized that MSCs preferentially migrate to the ischemic cortical region in the relatively early period after stroke, but then migrate to the ischemic striatal region in the relatively late period after stroke, and that any time-dependent differences in chemotactic signals after stroke might result in differential MSC migration. To better evaluate this hypothesis, an *in vitro* migration assay was performed. The results indicated that transwell migration of MSCs was more prominent with tissue extract from cortex obtained 1 day after MCAo than with extracts that were obtained at 4 and 7 days after MCAo. However, in the striatum, MSC migration was more prominent in tissue extracts obtained at 4 or 7 days after MCAo than in extracts obtained 1 day after MCAo (Fig 4). The migration assay results were consistent with the fluorescent imaging results which indicated that MSCs were primarily localized to the cortex in the D1 group and to the striatum in the D4 and D7 groups.

### MCP-1 and SDF-1 Concentrations in Ischemic Regions

**Fig 5:**
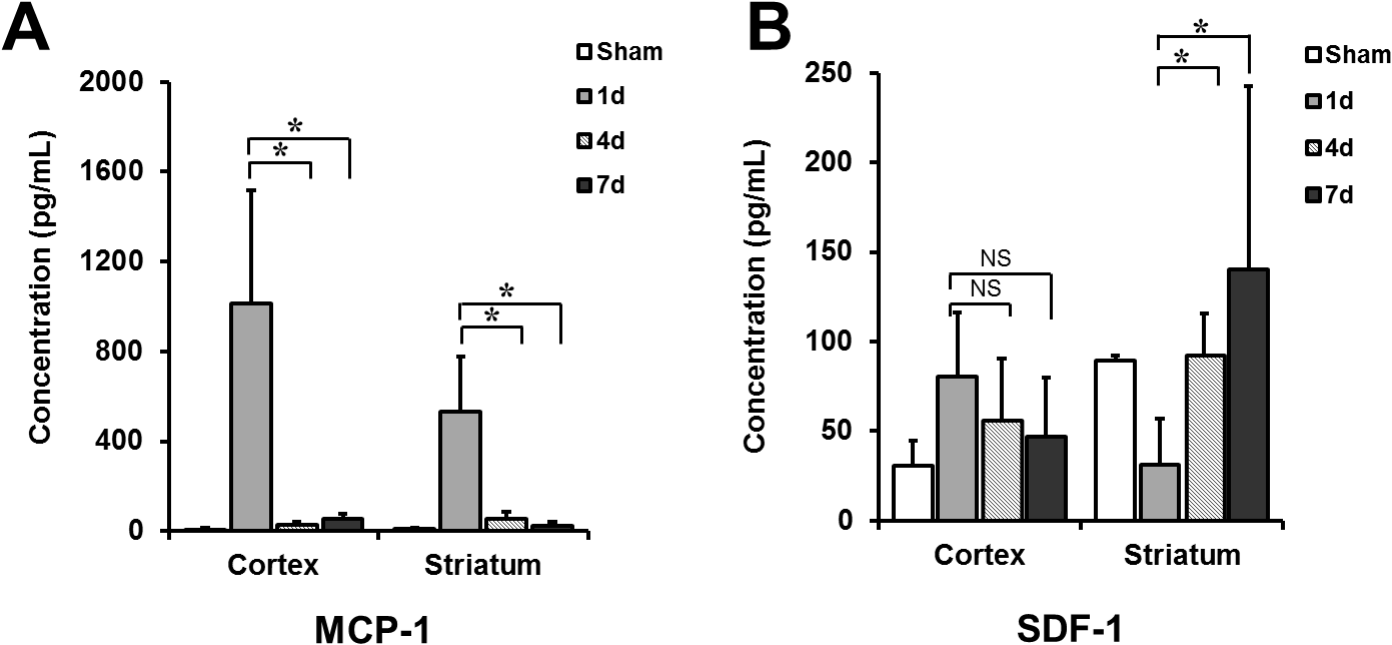
MCP-1 and SDF-1 expression levels in ischemic lesions according to the time after MCAo. (A) Expression level of MCP-1 was higher in brain extracts obtained from both cortex and striatum at 1 day after MCAo than in those at 4 or 7 days after MCAo (*P < 0.01). (B) In comparison, the SDF-1 concentration was higher at 4 or 7 days after MCAo than the concentration at 1 day after MCAo in striatum (*P < 0.01). However, there was no difference in the SDF-1 concentration according to the time from MCAo in cortex (n = 4 for each group). MCP-1 indicates monocyte chemotactic protein-1; SDF-1, stromal cell-derived factor-1; MCAo, middle cerebral artery occlusion.

The MCP-1 and SDF-1 concentrations were analyzed with an ELISA to investigate factors that could affect the differential migration of MSCs to ischemic regions based on the time of transplantation. The results showed that the level of MCP-1 was higher in both the cortex and striatum 1 day after MCAo than at 4 or 7 days after the MCAo. In comparison, the level of SDF-1 was higher 4 or 7 days after MCAo than 1 day after MCAo in the striatum. However, no difference was observed in the level of SDF-1 in the cortex after the MCAo procedure (Fig 5).

## Discussion

Although the timing of MSC transplantation may be one of the most important factors for achieving the best outcome after MSC therapy, there has been no consensus to date regarding the optimal time to transplant MSCs after stroke. Some studies have indicated that an immediate MSC transplantation following a stroke improved cerebral blood flow and blood-brain barrier permeability, and significantly reduced infarction volume [9,16]. However, in other studies, MSC administration 1 day, 7 days, or even at 1 month after stroke also had a positive effect on functional recovery, but it did not significantly decrease the area of infarction [7,10,17]. For this reason, the present study evaluated if there was a significant difference in the efficacy of MSC treatment based on the amount of time that elapsed between stroke and MSC transplantation. The results suggest that an earlier MSC transplantation may be associated with better functional recovery after stroke and are consistent with results of the recent studies addressing optimal timing for MSCs transplantation in stroke [18–20]. The efficacy of MSCs transplanted during the early period following a stroke may be associated with the immunomodulatory properties of MSCs rather than with neurogenesis or brain remodeling. This is because MSCs may decrease the inflammatory process induced by ischemia in the early period of a stroke. In addition, growth and trophic factors released from MSCs may also reduce cellular apoptosis after stroke [2,21–23]. In addition, the present study found that the preferential migration of MSCs to the ischemic cortical region, which modulates motor function. Our results also suggest that the time-dependent differential expression of MCP-1 and SDF-1 between ischemic regions could mediate the differential migration of MSCs.

Brain ischemia initiates an inflammatory reaction that produces various chemokines such as interleukin-8, MCP-1, SDF-1, and macrophage inflammatory protein-1. Specific chemokines, such as MCP-1 and SDF-1 have been suggested to act as key factors that stimulate MSC migration towards the ischemic region [24–26]. Previous studies demonstrated that the level of MCP-1 in the ischemic brain tissue extracts increased at 6 hours after MCAo, peaked at 48 hours, and decreased after 7 days. In addition, in regards to MSCs, the chemotactic activity of MCP-1 peaked at 24 hours after MCAo. In contrast, SDF-1 expression was transiently down-regulated at 6 hours, increased at 2 and 4 days, and then peaked within 7 days after ischemic injury [24,27]. These findings are consistent with the research findings of this study. However, in the recent study, the basic fibroblast growth factor and SDF-1 induced by cell transplantation play a role in MSC efficacy after stroke [20]. In the present study, MCP-1 and SDF-1 were differentially expressed over time in ischemic brain cortex and striatum after MCAo. MCP-1 levels were highest at one day of ischemia and appeared to play a pivotal role in the promotion of preferential MSC migration to the ischemic cortical region in the D1 group. In comparison, the MSC migration to the ischemic striatal region in the D4 and D7 groups appeared to be mainly supported by SDF-1. Although MCP-1 and SDF-1 levels were only measured in the present study, the data suggest that differential MSC migration after stroke could be mediated by the time-dependent differential expression of chemokines, including MCP-1 and SDF-1 after stroke. Specifically, the more prominent MSC migration to the cortex and better functional improvement seen in earlier MSC transplantation could be related to the increased expression of MCP-1 in the early phase after an ischemic stroke. Additional studies are needed to elucidate the time-dependent differential migration mechanism of MSCs.

Results of this study are in agreement with a few recent reports that investigated the optimal timing for MSC transplantation in consideration of the fact that an earlier transplantation could be associated with better outcomes than a later transplantation [19,28,29]. However, a few clinical studies which investigated the feasibility and efficacy of MSCs for stroke patients, used BM-derived, *ex vivo* expanded autologous MSCs for transplantation. It was not possible to transplant autologous MSCs within an optimal time window after the onset of stroke, since it required a minimum of several weeks to expand the MSCs beyond the target cell number [12,30]. Therefore, our results suggest that a clinical strategy using the allogeneic MSCs might be more optimal than the strategy using autologous MSCs for the stroke treatment. In general, MSCs are considered to have relatively low immunogenicity, in spite of constitutive HLA-class I expression and IFN-γ inducible HLA-class II expression [31]. Preclinical models and clinical trials using allogeneic MSCs have demonstrated that no adverse events were associated with allogeneic MSC transplantation [32,33]. Furthermore, several clinical studies that used third-party MSCs suggested that third-party MSC infusion is also feasible [32,34]. Altogether, these findings suggest that an early transplantation with allogeneic MSCs following a stroke could prove to be a better option for stroke treatment than delayed transplantation with autologous MSCs. In addition, another alternative option for early MSC transplantation would be to use autologous MSCs which were pre-cryopreserved after *ex vivo* expansion before the onset of stroke in patients who exhibited high-risk factors for stroke.

In conclusion, our results suggest that an earlier MSC transplantation could be associated with a better functional recovery after stroke, which can be explained by the preferential migration of MSCs to the ischemic cortical region which was observed in the group that received early transplantation. The time-dependent differential expression of MCP-1 and SDF-1 between ischemic regions (cortex vs. striatum) after stroke seemed to mediate the differential migration of the MSCs. Additional studies are needed to elucidate the optimal timing of transplantation and the optimal source of MSCs to achieve the best outcomes.
